# Gut Microbiota Composition in Male Rat Models under Different Nutritional Status and Physical Activity and Its Association with Serum Leptin and Ghrelin Levels

**DOI:** 10.1371/journal.pone.0065465

**Published:** 2013-05-28

**Authors:** María Isabel Queipo-Ortuño, Luisa María Seoane, Mora Murri, María Pardo, Juan Miguel Gomez-Zumaquero, Fernando Cardona, Felipe Casanueva, Francisco J. Tinahones

**Affiliations:** 1 Biomedical Research Laboratory, Virgen de la Victoria Hospital (FIMABIS), Malaga, Spain; 2 CIBER of Physiopathology of Obesity and Nutrition (CIBEROBN), Instituto de Salud Carlos III, Madrid, Spain; 3 Health Research Institute of Santiago de Compostela (IDIS), University Hospital Complex of Santiago de Compostela, Santiago de Compostela (CHUS/SERGAS), Spain; 4 Molecular Biology Laboratory, Civil Hospital (FIMABIS), Malaga, Spain; 5 Endocrinology and Nutrition Service, Clinical Hospital of Santiago de Compostela, Santiago de Compostela, Spain; 6 Endocrinology and Nutrition Service, Virgen de la Victoria Hospital, Malaga, Spain; Instutite of Agrochemistry and Food Technology, Spain

## Abstract

**Background:**

Several evidences indicate that gut microbiota is involved in the control of host energy metabolism.

**Objective:**

To evaluate the differences in the composition of gut microbiota in rat models under different nutritional status and physical activity and to identify their associations with serum leptin and ghrelin levels.

**Methods:**

In a case control study, forty male rats were randomly assigned to one of these four experimental groups: ABA group with food restriction and free access to exercise; control ABA group with food restriction and no access to exercise; exercise group with free access to exercise and feed ad libitum and ad libitum group without access to exercise and feed ad libitum. The fecal bacteria composition was investigated by PCR-denaturing gradient gel electrophoresis and real-time qPCR.

**Results:**

In restricted eaters, we have found a significant increase in the number of *Proteobacteria, Bacteroides, Clostridium, Enterococcus, Prevotella and M. smithii* and a significant decrease in the quantities of *Actinobacteria, Firmicutes, Bacteroidetes, B. coccoides-E. rectale group, Lactobacillus* and *Bifidobacterium* with respect to unrestricted eaters. Moreover, a significant increase in the number of *Lactobacillus, Bifidobacterium* and *B. coccoides–E. rectale* group was observed in exercise group with respect to the rest of groups. We also found a significant positive correlation between the quantity of *Bifidobacterium* and *Lactobacillus* and serum leptin levels, and a significant and negative correlation among the number of *Clostridium, Bacteroides* and *Prevotella* and serum leptin levels in all experimental groups. Furthermore, serum ghrelin levels were negatively correlated with the quantity of *Bifidobacterium, Lactobacillus* and *B. coccoides–Eubacterium rectale* group and positively correlated with the number of *Bacteroides* and *Prevotella*.

**Conclusions:**

Nutritional status and physical activity alter gut microbiota composition affecting the diversity and similarity. This study highlights the associations between gut microbiota and appetite-regulating hormones that may be important in terms of satiety and host metabolism.

## Introduction

Human eating disorders such as anorexia nervosa (AN) is an enormous public health problem in industrialized countries, and is characterized by extreme dietary restriction resulting in a sustained low weight [1]. Additionally, a significant proportion of AN patients shows evidence of abnormally high physical activity levels [2], [3]. It should be emphasized that AN is linked with a significant loss of adipose tissue and a decrease in energy metabolism [4]. On the other hand, there is no doubt that leptin plays an important role in AN, because leptin secretion is profoundly altered in this eating disorder [5]. Leptin is secreted by adipocytes and its primary role is to provide the central nervous system with a signal of the state of the body energy balance, which helps to control the appetite and food intake, and to maintain a stable body weight. Several studies have observed that serum leptin concentration is proportional to body fat mass during energy balance [6], and declines sharply in periods of energy deficit [7]. In addition, it has been found that patients with AN have higher fasting serum ghrelin levels than healthy subjects, which return to normal values after weight gain [8]. Ghrelin is mainly produced by the stomach but also by many other tissues [9]. Among the functions of ghrelin are the stimulation of the appetite and food intake, increasing fat mass deposition and weight gain and influencing glucose and lipid metabolism [10], [11]. The deregulation of all these mechanisms would be responsible for eating disorders like anorexia. Recently, it has been suggested that gut microbiota composition plays a role in the pathophysiology of eating disorders, since gut microbiota is able to partially mediate the appetite control regulating the level and type of autoantibodies targeting the appetite-regulating hormones [12], [13]. In addition, the host nutritional status may be markedly influenced by the composition and activities of the gut microbiota [14]. Moreover, several evidences indicate that the gut microbiota is involved in the control of host energy metabolism [15].

Activity-based anorexia (ABA) is a well established animal model used to study different aspects of AN and situations of undernutrition plus increased activity [16], [17]. In this rat model, anorexia is induced by restricting food intake to one daily feeding period (1–2 h) and permitting free access to a running wheel during the rest of the day (22–23 h). In this situation ABA rats exhibit a reduction in food intake, an increase in wheel activity and a progressive body weight decrease [16], [6].

The aim of the present study, therefore, was to characterize the composition of fecal microbiota in rat models under different nutritional status and physical activity in order to determine whether there were significant differences in the gut microbiota composition between these rat groups; if so, to quantify the differences and to identify the possible association of the gut microbiota found in these rat models with their serum leptin and ghrelin levels.

## Materials and Methods

### Ethics Statement

The animal studies were conducted in accordance with the ethical guidelines for the care and use of laboratory animals of the National Institutes of Health. All procedures were approved by the Animal Care ethics Committee of Santiago de Compostela University (Santiago de Compostela, Spain).

### Animal models

Forty Male Sprague Dawley rats (160 g/5 weeks old) (Charles River Breeding Laboratory, Raleigh, NC) from the same bred were housed in a temperature-controlled room with a 12-h light/12-h dark cycles with free access to standard chow diet and water. A case-control study was performed. After 5 days acclimatization weight-matched animals were randomly assigned to one of four experimental groups (10 rats by group): (a) Activity based anorexia (ABA) group: rats starved by restricting food access to 23 hours per day and confined to running wheels except during a 60 min meal per day, (b) Control ABA group: rats submitted to the same food restriction schedule as ABA with no wheel access exercise, (c) Exercise group: rats feed ad libitum with free access to the activity wheel and (d) Ad libitum group: rats feed ad libitum but without access to the activity wheel. The ABA group was performed following previously established model by Routtenberg and Kuznesof [16]. For ethical reasons, ABA rats were not allowed to lose more than 20–30% of their initial body weight. All animals were housed individually in custom-designed, stainless steel cages, which in the running groups were connected to running wheels (16 cm in diameter) coupled to a turn counter (Harvard Apparatus, MA). Food intake, wheel running and body weight were measured daily. These rats were sacrificed by decapitation after 6 days of experiment and blood samples, tissues and fecal content were collected. The 6-day experimental length was fixed as it is the time where ABA rats reach an approximately 30% body weight reduction and some of them started to show gastric ulcers.

### Hormone determination

Blood samples collected at the end of the study after the rats were sacrificed were immediately centrifuged and total ghrelin and leptin levels were determined in serum by a double antibody RIA using kits provided by Linco Research (St Charles, MI) as previously described [18], [19]. The limits of sensitivity of the assays were 100 pg/ml for ghrelin, 0.5 ng/ml for leptin, and 0.1 ng/ml for insulin.

### DNA extraction from fecal samples

Fecal samples were immediately kept after collection at −80°C and stored until analyzed. DNA extraction from 200 mg of stools was performed using the QIAamp DNA stool Mini kit (Qiagen, Hilden, Germany) following the manufacturer’s instructions. The DNA concentration was determined by absorbance at 260 nm (A260), and the purity was estimated by determining the A260/A280 ratio with a Nanodrop spectrophotometer (Nanodrop Technologies, Wilmington, DE).

### Analysis of fecal microbiota by PCR-DGGE

Fecal samples from each subject were examined by determining PCR-DGGE profiles as recently published by us [20]. The V2–V3 regions of the 16S rRNA genes (positions 339 to 539 in the *Escherichia coli* gene) of bacteria in the fecal samples were amplified by primers HDA1-GC (5′-**CGC CCG CCG CGC GCG GCG GGC GGG GCG GGG GCA CGG GGG G**CC TAC GGG AGG CAG CAG T-3′; (the GC clamp is in boldface) and HDA2 (5′-GTA TTA CCG CGG CTG CTG GCA C-3′) generating a 200 bp product. Aliquots (2 µL) of DNA were amplified by real-time PCR (20 µL final volume) in a 7500 Fast Real-Time PCR Systems instrument using Fast SYBR Green Master Mix and 200 nM of each of the universal primers HDA1-GC/HDA2 with the following amplification program: initial denaturation was at 95° for 20 s, amplification was carried out using 45 cycles including denaturation at 95°C for 3 s, annealing at 55°C for 30 s and extension at 72°C for 1 min.

After real time PCR 15 µL of products were mixed with 6 µL loading dye before loading. Electrophoresis was performed with a DCode ™ Universal Mutation Detection System instrument (Bio-Rad). 6% polyacrylamide gels were prepared and electrophoresed with 1× TAE buffer prepared from 50× TAE buffer (2 M Tris base, 1 M glacial acetic acid, 50 mM EDTA). The denaturing gradient was formed by using two 6% acrylamide (acrylamide/bisacrylamide ratio 37.5∶1) stock solutions (Bio-Rad). The gels contained a 20-80% gradient of urea and formamide that increase in the direction of electrophoresis. Electrophoretic runs were in a Tris-acetate-EDTA buffer (TAE 1x) (40 mmol/L Tris, 20 mmol/L acetic acid, and 1 mmol/L EDTA, pH 7.4) at 130 V and 60°C for 4.5 h. Electrophoresis was stopped when a xylene cyanol dye marker reached the bottom of a gel. Gels were stained with ethidium bromide (0.5 mg/L) for 5 min, rinsed with deionized water, viewed by UV transillumination and photographed with Gelcapture image acquisition software (DNR Bio-Imaging Systems Ltd). All the samples were analyzed on the same DGGE run to avoid the possible influence of variations in electrophoretic conditions between different runs. Similarities between banding patterns in the DGGE profile were calculated based on the presence and absence of bands and expressed as a similarity coefficient (Cs). Gels were analyzed using BioNumerics software (Applied Maths, Sint-Martens-Latem, Belgium). Normalized banding patterns were used for cluster analysis. The Dice similarity coefficient was used to calculate pairwise comparisons of the DGGE fingerprint profiles obtained. A Cs value of 100% indicates that DGGE profiles are identical while completely different profiles result in a Cs value of 0%. The UPGMA (unweighted pair group method with arithmetic mean) algorithm was used for construction of dendrograms.

### Sequencing of selected bands from DGGE gels

Bands of specific interest were excised from DGGE gels with a sterile razor, placed in 40 µl sterile water and incubated at 4°C for diffusion of DNA into the water. DNA were used in a second PCR with HDA1/2 primers without GC-clamp (initial denaturation 95° for 20 s, followed 45 cycles including denaturation at 95°C for 3 s, annealing at 55°C for 15 s and extension at 72°C for 10 s). Subsequently, the PCR products will be directly cloned into pCR® 4-TOPO (Invitrogen, UK) according to the manufacturer's instructions. Plasmid DNA will be isolated from the cells using the Qiagen Mini Spin Prep kit (QIAGEN, Germany), and subjected to PCR (HDA1/2-GC) as earlier described. PCR products were diluted until 20 ng/μL, purified with ExoSAP-IT (USB corporation, Miles Road, Cleveland, Ohio, USA) and sequenced in an ABI 3130 (Applied Biosystems, USA) using the BigDie-Kit-Standard. Nucleotide sequence data obtained were analyzed using MicroSeqID v2.1.1 software (Applied Biosystems, USA).

### Microbial quantification by real-time PCR

Specific primers targeting different bacterial genera were used to characterize the fecal microbiota by real-time qPCR (Table 1) [21]–[28]. Briefly, real-time qPCR experiments were performed with a LightCycler 2.0 PCR sequence detection system using the FastStart DNA Master SYBR Green kit (Roche Diagnostics, Indianapolis, IN). All PCR tests were carried out in duplicate with a final volume of 20 µL containing 1 µL of each fecal DNA preparation and 200 nM of each primer (Table 1). The thermal cycling conditions used were as follows: an initial DNA denaturation step at 95°C for 10 min, followed by 45 cycles of denaturation at 95°C for 10 s, primer annealing at optimal temperature (Table 1) for 20 s, extension at 72°C for 15 s. Finally, melt curve analysis was performed by slowly cooling the PCRs from 95 to 60°C (0.05°C per cycle) with simultaneous measurement of the SYBR Green I signal intensity. Melting-point-determination analysis allowed the confirmation of the specificity of the amplification products.

**Table 1:** Primers used for real-time PCR.

Target group	Oligonucleotide sequence (5′–3′)	Reference	Amplicon size (bp)
*Bacteroidetes*	CATGTGGTTTAATTCGATGAT	(21)	126
	AGCTGACGACAACCATGCAG		
*Bacteroides*	GAGAGGAAGGTCCCCCAC	(21)	106
	CGCTACTTGGCTGGTTCAG		
*Lactobacillus*	GAGGCAGCAGTAGGGAATCTTC	(22)	126
	GGCCAGTTACTACCTCTATCCTTCTTC		
*Fusobacteium*	CCCTTCAGTGCCGCAGT	(23)	273
	GTCGCAGGATGTCAAGAC		
*Firmicutes*	ATGTGGTTTAATTCGAAGCA	(21)	126
	AGCTGACGACAACCATGCAC		
*Actinobacteria*	CGCGGCCTATCAGCTTGTTG	(24)	600
	CCGTACTCCCCAGGCGGGG		
*Bifidobacterium*	CTCCTGGAAACGGGTGG	(25)	550
	GGTGTTCTTCCCGATATCTACA		
*Prevotella*	GGTTCTGAGAGGAAGGTCCCC	(26)	121
	TCCTGCACGCTACTTGGCTG		
*Enterococcus*	CCCTTATTGTTAGTTGCCATCATT	(27)	144
	ACTCGTTCTTCCCATGT		
*Proteobacteria*	CATGACGTTACCCGCAGAAGAAG	(23)	195
	CTCTACGAGACTCAAGCTTGC		
*Clostridium*	GCACAAGCAGTGGAGT	(25)	239
Cluster IV	CTTCCTCCGTTTTGTCAA		
*Blautia coccoides*–*Eubacterium rectale* group	CGGTACCTGACTAAGAAGC AGTTTCATTCTTGCGAACG	(27)	429
*Methanobrevibacter smithii*	CCGGGTATCTAATCCGGTTC CTCCCAGGGTAGAGGTGAAA	(28)	123

The bacterial concentration from each fecal sample was calculated by comparing the *C*t values obtained from the standard curves with the LightCycler 4.0 software. Standard curves were constructed for each experiment using serial tenfold dilutions of bacterial genomic DNA (of known concentration) from pure cultures, corresponding to 10^1^–10^10^ 16S rRNA gene copies/ gram of feces. The mass for one bacterial genome was calculated by using the Avogadro constant and assuming the mean molecular weight of a base pair to be 660 g/mol. Standard curves were normalized to the copy number of the 16S rRNA gene for each species. For the species whose copy number of 16S rRNA operon was not published, it was calculated by averaging the operon numbers of the closest bacterial taxa from the ribosomal RNA database rrnDB [29]. The 16S rRNA gene copies in each sample were normalized to gram of feces. The different strains used were obtained from the Spanish Collection of Type Cultures (CECT) (*Bacteroides vulgatus* NCTC 11154, *Fusobacterium varium* NCTC 10560, *Enterococcus faecalis* CECT 184, *Enterobacter cloacae* CECT 194, *Clostridium perfringens* CECT 376) and the American Type Culture Collection (ATCC) (*Bifidobacterium bifidum* ATCC 15696, *Lactobacillus casei* ATCC 334D-5, *Prevotella intermedia* ATCC 25611D-5, *Ruminococus productus*, ATCC 27340D-5, *Methanobrevibacter smithii* ATCC 35061).The data presented are the mean values of duplicate qPCR analyses.

### Statistical analysis

Results are expressed as mean values and standard deviations. The statistical analysis was performed with SPSS 15.0 software (SPSS Inc., Chicago, IL). The 16S rRNA gene copies values were converted into logarithmic values before the statistical analysis. The Kruskal-Wallis test was used to check changes in bacterial number and the biochemical variables between the groups of rats. The Mann-Whitney U test with Bonferroni *Post-hoc* test was used to compare one group of rats to each other. The Spearman correlation coefficient was calculated to estimate the linear correlations between variables. A multivariate regression analysis was performed to identify independent predictors for serum leptin and ghrelin levels. In this analysis, bacterial group, weight, food intake and activity were selected as independent variables. Barnard's exact unconditional test was used to compare the proportions of one group of rats to each other. Statistical significance was set at a *P* value of <0.05. All data are presented in the text as the mean ± SD.

## Results

### Exercise, dietary aspects and appetite-regulating hormones levels

**Figure 1:**
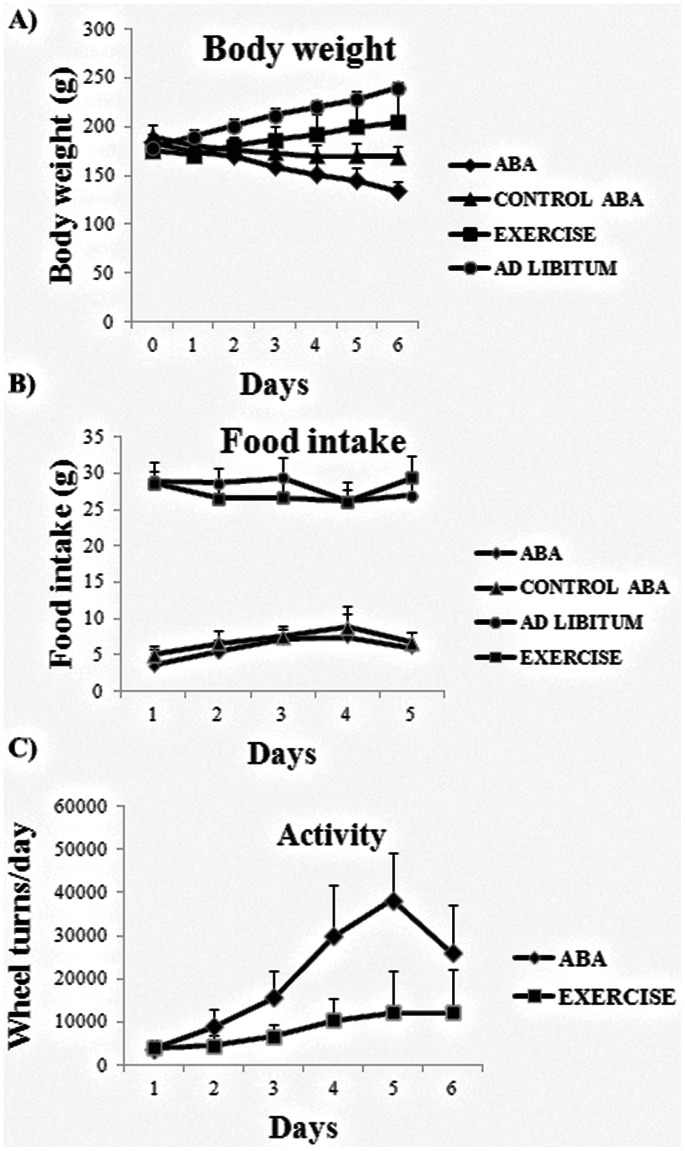
Representation of the relative daily body weight variation (A) and daily food intake (B) in the all study groups (N =  10 rats per group). The figure (C) shows a 23 h registered activity in ABA and exercise groups.

As figure 1A shows, our results confirmed that exercise and ad libitum groups of rats showed a progressive increase in body weight and significant differences were found between them. In contrast, feeding restriction to 1 h per day (control ABA group), and particularly the combination of this fasting regime with exercise (ABA group), significantly reduced body weight with respect to exercise and ad libitum groups from days 1 to 6. While, the control ABA group, showed a decrease in body weight that was stabilized from day 3 to 6 (figure 1A). With regard to food intake, we found no significant variations in daily food intake between exercise and ad libitum groups and between ABA and control ABA groups, respectively (figure 1B). Finally, when measuring the activity we found that the exercise group showed significantly less wheels turns than ABA group from days 3 to 5 (figure 1C).

**Figure 2:**
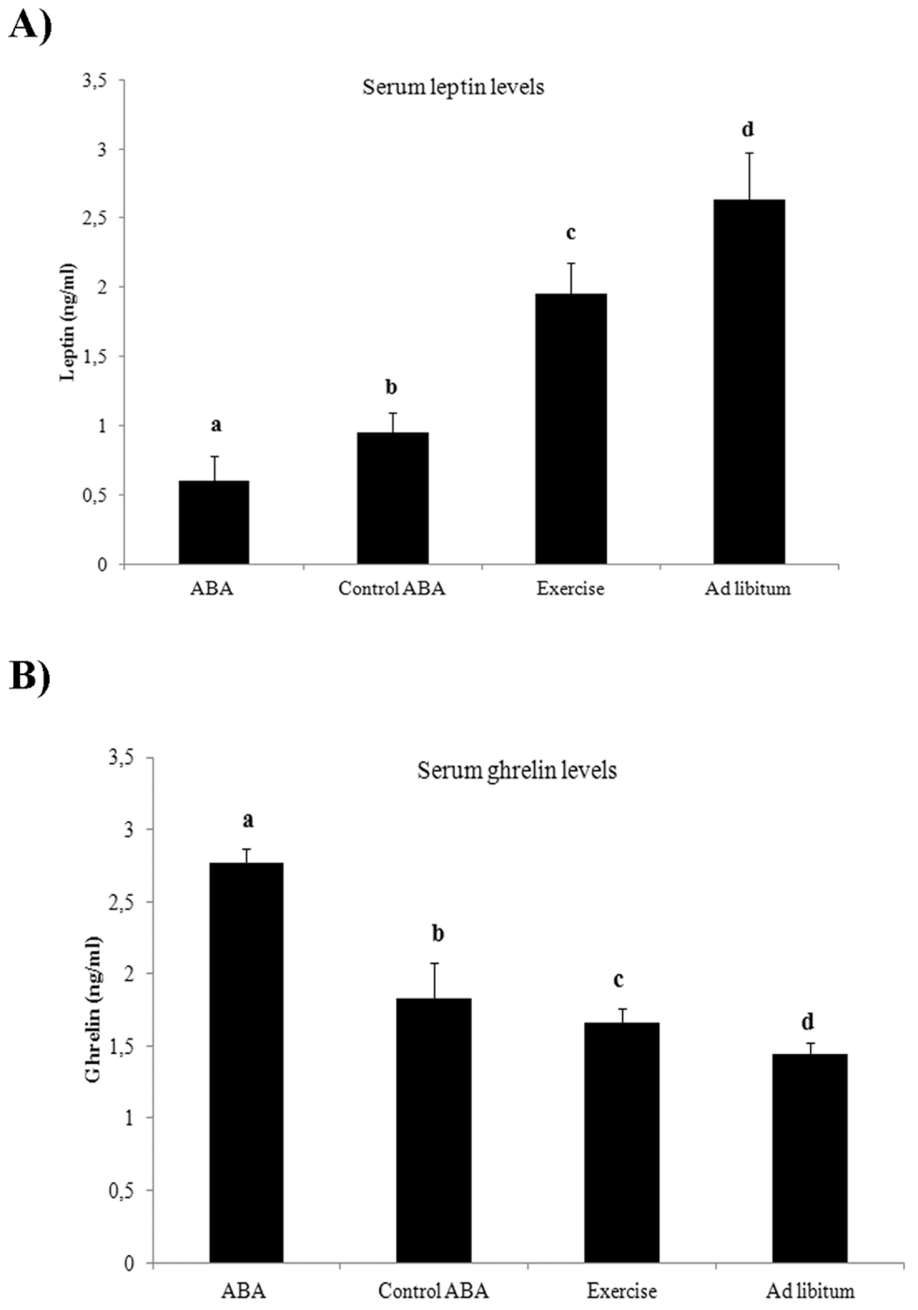
Serum leptin (A) and grhelin (B) levels (C) at day 6 of study in all rat groups (N =  10 rats per group). The Mann-Whitney U test was used to compare a rat group to each other. Different superscript letters are significantly different *P*<0.05 (Bonferroni *Post-hoc* test).

On the other hand, we observed a significant serum leptin decrease and ghrelin increase

in both ABA and control ABA compared to exercise and ad libitum. Moreover, we have found significant differences between the ABA and control ABA groups and between exercise and ad libitum groups in the serum leptin and ghrelin levels (figure 2A and 2B).

### PCR-DGGE fingerprint analysis and bacterial band identification in the fecal samples

Variations were found in the presence or absence (qualitative) and intensity (quantitative) of the bands between rat groups in the generated host-specific fingerprints. DGGE band profiles showed differences in band richness between the four groups. Analyzing the diversity of microbiota, we have found that the mean of DGGE bands was 11.16±2.0 for ABA, 13.50±1.04 for control ABA, 15.66±1.21 for exercise and 19.0±1.41 for ad libitum groups. Moreover, these differences in band richness were significant between all groups (P<0.05). On the other hand, some bands were seen in fingerprints from all the rats (in different lane but at the same position), indicating that specific species of the predominant microbiota were common to all rat groups.

**Figure 3:**
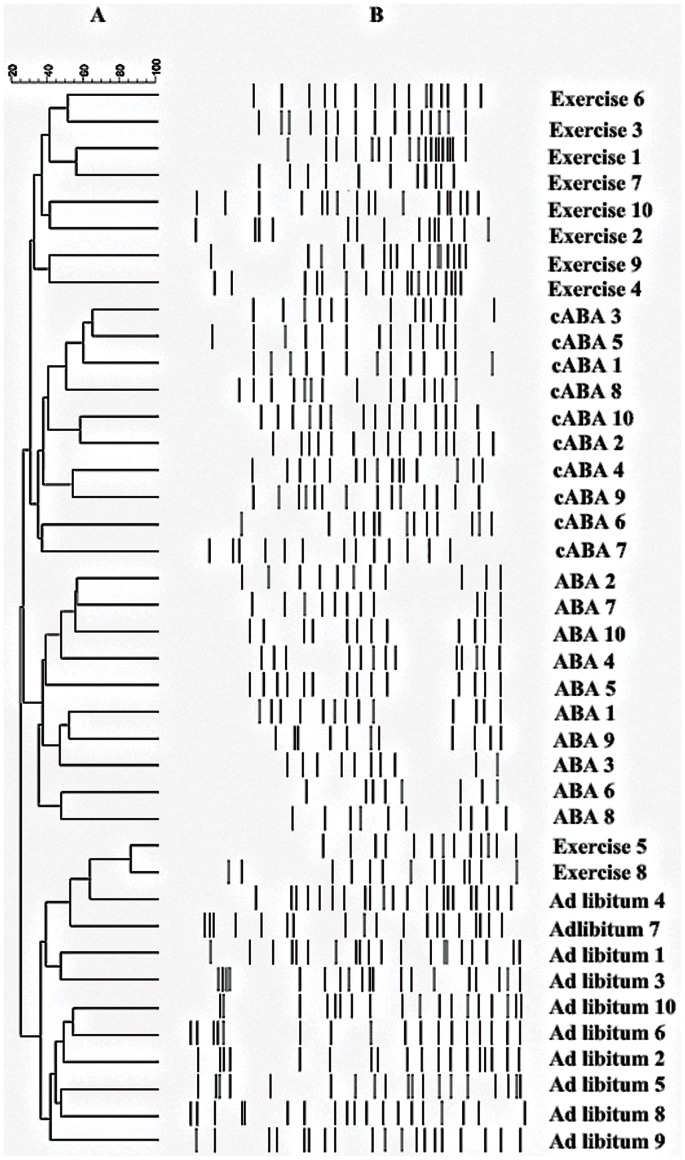
Dendrograms of electrophoretic band patterns obtained in the denaturing gel gradient electrophoresis experiment with universal primers in the fecal samples collected from ABA, control ABA (cABA), exercise and ad libitum rats. A: Cluster analysis; B: DGGE profiles of fecal samples.

The Dice similarity coefficient was used to calculate the similarity index of the DGGE band profiles for the four rat groups. The means of similarity index were 35.67%, 37.93%, 42.57% and 49.37% for ABA, control ABA, exercise and ad libitum groups respectively. The mean inter-group similarity index between ABA and control ABA groups was 25.75%, between exercise and ad libitum groups was 30.51%, between ABA and exercise group was 21.22% and between control ABA and ad libitum was 24.77%, which was fewer than the intra-group similarity index for the four rat groups above described. The intensity and position of DGGE detected bands were subjected to cluster analysis. The dendrogram shows four large clusters of feeding restriction plus exercise rats, feeding restriction rats, exercise rats and ad libitum rats, except for two exercise rats belonging to the cluster of ad libitum rats (Figure 3).

All the bands from all rat profiles in the different groups were cloned and sequenced to identify the dominant microbiota and the sequence similarity matches for bands were analyzed by MicroSeqID v2.1.1 software. Bacterial identification showed that the majority of bacteria represented in our fingerprints corresponded to four phyla (Table 2). Most of the sequences belonged to *Firmicutes* and *Bacteroidetes,* with the rest distributed among *Actinobacteria* and *Proteobacteria*. Nevertheless, we also observed important differences between all rat groups in the frequencies of different genus within these phyla. In the ABA and ad libitum groups, we have found an increase in the frequencies of *Bacteroides, Prevotella, Clostridium* and *Helicobacter* with respect to the control ABA and exercise groups respectively. Finally, with respect to the frequencies of *Lactobacillus* and *Bifidobacterium*, we observed a significant decrease in the ABA group with respect to exercise group (p =  0.008 and p = 0.05 respectively). In addition, a significant decrease was also found between exercise and ad libitum groups (p = 0.05) (Table 2).

**Table 2:** Bacterial identification after the sequencing of the bands cloned from the DGGE analysis of fecal samples from the all rat groups.

Type bacteria genus(sequencing results ofthe bands)	ABA group^a^n = 45	Control ABA group^a^n = 35	Exercise group^a^n = 34	Adlibitum group^a^n = 46	Sequencesimilarity (%)
**Phylum ** ***Bacteroidetes***					
Genus *Bacteroides*	13 (28.8%)^a^	10 (28.6%)^a^	8 (23.5%) a	12 (26%) a	99.86
Genus *Prevotella*	14 (28.6%) a	10 (28.6%) a	7 (20.58%)^a^	13 (28.3%)^a^	99.91
**Phylum ** ***Firmicutes***					
Genus *Lactobacillus*	0a, c	1 (2.8%) a, b,c	5 (14.7%)^b^	1 (2.2%)a,c	99.73
Genus *Clostridium*	15 (33.3%)	11 (31.4%)	9 (26.5%)	16(34.8%)	99.99
**Phylum ** ***Actinobacteria***					
*Genus Bifidobacterium*	0^a^	1 (2.8%) a, b	3(8.8%)^b^	1 (2.2%)a,b	99.92
**Phylum ** ***Proteobacteria***					
Genus *Helicobacter*	2 (4.4%) a	1 (2.8%) a	1 (2.9%) a	2 (5.3%) a	99.65
Genus *Campylobacter*	1 (2.2%) a	1 (2.8%) a	1 (2.9%) a	1 (2.2%) a	99.73

a Refers to the frequency (and percent) of each unique bacteria genus in the ABA or Control ABA or exercise or ad libitum group.

"n" number of bands cloned, sequenced and identified in each rat group.

N = 10 rats per group. Barnard's exact unconditional test was used to compare the proportions of one group of rats to each other.

Values in a row with different superscript letters are significantly different *P*<0.05.

### Comparative analysis of gut microbiota communities in the rat models under different nutritional status and physical activity

Changes in the bacterial population abundance were assessed in the fecal samples of all groups of rats both at phylum and genera levels (Tables 3). We found a significant decrease in the number of *Actinobacteria* phylum between the ABA group and the rest of the study groups. In addition, a significant increase in the number of *Proteobacteria* and a significant decrease in the quantity of *Bacteroidetes* and *Firmicutes* were observed in the ABA with respect to exercise and ad libitum groups. Finally, the quantity of *Actinobacteria* and *Bacteroidetes* was significantly increased while the number of *Firmicutes* was significantly decreased in exercise group compared to ad libitum group.

**Table 3:** Real-time PCR quantification of microbiota phyla, genera, groups and species in the study groups of rat.

	ABAgroup	Control ABAgroup	Exercisegroup	Ad libitum group	*P*
***Proteobacteria***	7.14±0.84^a^	6.83±0.69^a^	5.15±0.18^c^	5.16±0.28^ c^	0.001
***Fusobacteria***	6.42±0.26^a^	6.31±0.16^a^	6.40±0.49 ^a^	6.36±0.59^ a^	0.180
***Actinobacteria***	5.91±0.99^a^	7.41±0.45^b^	8,33±0.01^ c^	7.84±0.20^ d^	0.013
*Bifidobacterium*	6.75±1.07^a^	6.84±1.2^a^	9.33±0.10^ c^	7.97±0.37^d^	0.003
***Bacteroidetes***	9.24±0.22^a^	9.43±0.18^a^	9.82±0.14 ^c^	9.68±0.24^ c^	0.037
*Bacteroides*	7.95±0.13^a^	7.64±0.48^ b^	6.12±0.01^ c^	7.10±0.38^ d^	0.001
*Prevotella*	8.85±0.50^a^	8.04±0.03^b^	7.17±0.08^ c^	7.45±0.13^ d^	0.001
***Firmicutes***	7.24±0.33^a^	7.33±0.15^a^	7.63±0.22^ c^	8.80±0.35^ d^	0.007
*B. Coccoides- E. rectale group*	7.34±1.23^a^	7.23±0.98^a^	9.74±1.14^c^	8.55±0.92^ d^	0.001
*Enteroccocus*	8.41±1.73^a^	8.35±1.8^a^	4.08±0.66^c^	6.55±1.7^ d^	0.002
*Clostridium*	5.30±0.16^a^	5.10±0.2^b^	4.39±0.06^ c^	4.87±0.14^ d^	0.001
*Lactobacillus*	6.20±0.10^a^	6.29±0.15^a^	7.69±0.16^ c^	6.91±0.21^ d^	0.033
***Euryarchaeota*** *M. smithii*	7.95±0.99^ a^	7.89±1.07^ a^	6.32±1.02^ c^	6.43±1.10^ c^	0.001

Values are presented as means ± SD and expressed as log_10_ 16S rRNA gene copies/gram of feces. N = 10 rats per group. The *P* value in the last column was based on the Kruskal-Wallis test. The Mann-Whitney U test was used to compare a rat group to each other. Values in a row with different superscript letters are significantly different *P*<0.05 (Bonferroni *Post-hoc* test).

Within *Firmicutes*, the number of *Clostridium* was significantly increased in the ABA group with respect to the control ABA and the exercise and ad libitum groups. In addition, we found a significant increase in the number of *Enteroccocus* accompanied by a significant decrease in the quantities of *B. Coccoides-E rectale* group and *Lactobacillus* in the ABA and control ABA groups with respect to the exercise and ad libitum groups. On the other hand, the quantity of *B. Coccoides-E rectale* group and *Lactobacillus* showed a significant increase while the number of *Clostridium* and *Enteroccocus* presents a significant decrease in the exercise group with respect to ad libitum group.

Within *Bacteroidetes*, we have found a significantly higher quantity of *Bacteroides* and *Prevotella* in the ABA group with respect to control ABA, exercise and ad libitum group. In addition, a significant decrease in the number of *Prevotella* and *Bacteroides* was observed in the exercise group with respect to ad libitum group. Moreover, within *Actinobacteria* a significant decrease in the number of *Bifidobacterium* was observed in the ABA, control ABA and ad libitum groups with respect to exercise group. Finally, within *Euryarchaeota* a significant increase in the quantity of *M. smithii* in the ABA and control ABA with respect to exercise and ad libitum group has been found.

### Relation between gut microbiota composition and serum leptin and ghrelin levels

Moreover, we found a significant univariate correlation between the quantity of specific bacterial groups and the serum leptin and ghrelin levels. The analysis showed a significant positive correlation between the quantity of *Bifidobacterium* (r = 0.429, *P* <0.05) and *Lactobacillus* (r =  0.466, *P*<0.05) and serum leptin levels, and a significant and negative correlation among the number of *Clostridium* (r =  –0.677, *P*<0.001), *Bacteroides* (r =  –0.531, *P*<0.001) and *Prevotella* (r =  –0.885, *P*<0.001) and serum leptin levels in all the studied population. Furthermore, serum ghrelin levels were negatively correlated with the quantity of *Bifidobacterium* (r =  –0.496, *P*<0.05), *Lactobacillus* (r =  –0.499, *P*<0.05) and *B. coccoides–Eubacterium rectale* group (r =  –0.628, *P* = 0.001) and positively correlated with the number of *Bacteroides* (r =  0.529, *P*<0.05) and *Prevotella* ( =  0.686, *P*<0.001). A multivariate regression analysis that included the weight, food intake, activity and all the bacterial groups analyzed as independent variables, showed that only the increase in the number of *Lactobacillus* and *Bifidobacterium* (R^2^ = 0.989; *P*<0.001, β = 0.563 and *P*<0.05, β = 0.979 respectively) and the decrease in *Prevotella* and *Clostridium* (R^2^ = 0.989;*P*<0.05, β = –0.241 and *P*<0.05, β = –0.448 respectively) were associated with the serum leptin level. While the serum ghrelin level was associated with the decrease in the quantity of *Bifidobacterium, Lactobacillus* and *B. coccoides-E rectale* group (R^2^ = 0.984; *P* = 0.001, β = –0.400; *P*<0.05, β = –0.247 and *P*<0.001, β = –0.578 respectively) and the increase in the number of *Prevotella* (R^2^ = 0.984; *P*<0.001, β = 0.446).

## Discussion

In the present study we found that the nutritional status (food restriction and ad libitum regimens) and physical activity affected the composition of the gut microbiota. The food intake and activity present in our experimental rat models followed similar patterns as described previously [30], [31], however the gut microbiota composition of these animals has not been studied previously. In our study, to reduce variation in microbiota based in genetics, age and sex, male rats with similar weight from the same bred were acquired. Moreover, they were single housed to avoid competition for food as a source of variation. In this work, we have observed that rats with activity-based anorexia (ABA) develop hyperactivity and consume less food than rats with limited access to food and without access to running wheel (control ABA), since these rats adapt their feeding behavior to the shorter period of food availability. In presence of exercise, unrestricted eaters (exercise group) significantly increased the food intake compared to restricted eaters (ABA group), possibly because the exercise is more effective in creating a negative energy balance. In addition, the significant body weight reduction due to food restriction with access to exercise (ABA rats) was accompanied by a significant enhancement in the reduction of serum leptin concentration and a significant increase in the serum ghrelin levels, which reinforce the function of leptin as a starvation signal [32]. Moreover, this data suggest that low leptin levels could represent the key signal that triggers hyperactivity [33].

The analysis of the PCR-DGGE banding profiles showed that bacterial diversity was significantly lower in the two groups of rats with feeding restriction, especially in the ABA model. This lower bacterial composition reduced the set of ecosystem processes available in this bacterial community that may lead to less healthy animals [34]. In addition, the fecal DGGE analysis revealed a significantly lower intra-group similarity index in the restricted eaters groups (ABA and control ABA) than in the unrestricted eaters groups (exercise and ad libitum). Moreover, these intra-group similarity indexes of all rat groups were significantly higher than the inter-group similarity indexes, showing cluster of banding patterns characteristic for each rat group. These data suggest that the nutrition status and exercise may affect the diversity and similarity of the gut microbiota community present in this study models.

Sequencing results of the DGGE bands cloned also revealed differences in the microbiota composition between all the investigated groups. The sequence analysis of DGGE bands showed that the gut microbiota of the four rat groups was predominately composed of *Firmicutes* and *Bacteroidetes*. However, major differences were observed in the frequency of each genus of bacteria. We have observed differences in the frequency of bacteria in the ABA and ad libitum groups with respect to control ABA and exercise groups. These results suggest that the dominant microbiota genera could be modulated by both the feeding restriction and exercise.

Additional analyses with real-time qPCR were performed to obtain a quantitative estimation of the changes in the gut microbiota between the four experimental groups. We found significant differences between groups with or without feeding restriction at both phylum and genus level. Thus, we have detected a significant increase in the number of *Proteobacteria, Bacteroides, Clostridium, Enteroccoco, Prevotella* and *M. smithii* and a significant decrease in the quantity of *Actinobacteria, Firmicutes, Bacteroidetes, B. coccoides-E. rectale* group, *Lactobacillus* and *Bifidobacterium* in the restricted eaters with respect to the unrestricted eater groups. This distribution of gut microbiota among groups with and without feeding restriction may be linked to different nutrient availability in the gut. In accordance with us, Armougon *et al*. found that anorexic patients showed a significantly more quantity of *M. smithii* than lean patients. One explanation for these results may be associated with an adaptative attemp towards optimal exploitation of the very low caloric diet absorbed by these patients (35). Then, the increase of *M. smithii* in our restricted eaters may lead to optimization of food transformation in very low calorie diets.

It has been previously described that feeding restriction (fasting situation) stimulates the growth of the mucin degrading bacteria because these bacteria present a competitive advantage during nutrient deprivation [36], [37]. We have observed a significant increase in the number of *Prevotella* (which are responsible for the degradation of the intestinal mucin) in the ABA and control ABA with respect to exercise and ad libitum situations. This increase in *Prevotella* in the restricted eaters could involve an increase in the degradation and the lack of mucin (a glycoprotein produced by the host that maintains the integrity of the gut epithelium against pathogenic microorganisms as well as chemical, physical or enzymatic damage) on the epithelial layer of the gut, which would lead to a significant alteration in intestinal permeability. Increased mucosal permeability has previously reported by Sonoyama *et al* in fasted hamster due to the significant increase of *Akkermansi muciniphila*, a mucin degrading bacteria [38]. On the other hand, in these ABA and control ABA groups we have found a significant increase in the quantity of *Bacteroides* and *Clostridium* with respect to exercise and ad libitum groups. Based on cross-feeding mechanism among different bacterial groups, *Clostridium* spp. is able to utilize lactate and to convert it into acetate and propionate, and acetate is also produced by nearly all species of *Bacteroides*; however, these short fatty acids do not induce mucin synthesis [39]. Acetate was reported to protect against diet-induced obesity and had no acute effect on gut hormones while propionate was shown to induce gut hormones and reduce food intake [40]

Previous studies have described that weight reduction affects the composition of the gut microbial community in both mice and humans [41]–[43]. Moreover, it has been suggested that the effects of body weight changes on the gut microbiota may be mediated, in part, by changes in circulating leptin concentrations [32] since the level of this hormone may affect the composition of the gut microbiota by the stimulation of mucin production in the intestine [44], [45]. Accordingly, we have observed a connection between serum leptin concentrations and the composition of the gut microbiota. Thus, we have found that quantity of several bacterial genus is significantly correlated with serum leptin level. For instance, quantity of *Bifidobacterium, Lactobacillus*, and serum leptin concentrations is positively correlated, whereas the number of *Clostridium, Bacteroides* and *Prevotella* was negatively correlated with serum leptin level. Then, the significant decrease in body weight together with the significantly lower serum leptin levels found in the ABA and the control ABA animals with respect to exercise and ad libitum groups could be used to explain the significant changes found in the gut microbiota between these groups.

On the other hand, in our study we have observed a significant increase in the number of *Lactobacillus, Bifidobacterium* and *Blautia coccoides–Eubacterium rectale* group in the exercise group with respect to the ABA, control ABA and ad libitum groups. Both *Bifidobacteria* and *Lactobacillus* have the capacity to produce the organic acid lactate, which is converted into butyrate by butyrate-producing bacteria in the gut [46]. Barcenilla *et al*. showed that most of the butyrate-producing isolates from human fecal samples are related to the *Blautia coccoides–Eubacterium rectale* group [47]. Matsumoto *et al*. using exercise and sedentary Wistar rats suggested that exercise altered the composition of the microbiota in the cecum and increased the concentration of n-butyrate in the cecal content of the exercise rats [48]. Moreover, previous studies have shown that butyrate induces mucin synthesis [39], decreases bacterial transport across the epithelium [49], improves gut integrity by increasing tight junction assembly [50] and reduces serum ghrelin levels [40]. In our study we have found a significantly lower serum ghrelin level in the exercise and ad libitum groups with respect to ABA and control ABA groups. Furthermore, the serum ghrelin levels showed a significant negative correlation with the quantity of *Bifidobacterium, Lactobacillus* and *Blautia coccoides–Eubacterium rectale* group, and a significant positive correlation with the number of *Bacteroides* and *Prevotella*. Other authors have previously described the associations here found between fecal bacterial families and blood hormones, such as leptin and ghrelin in kittens under a protein/carbohydrate dietary intervention.They also found a positive association between *Lactobacillus* and blood leptin levels, but unlike us, the correlation between *Bifidobacteriaceae* and blood ghrelin levels was positive (51). These data suggest a role of the gut microbiota on satiety control.

In conclusion, these findings indicated that differences in the nutritional status and exercise alter the gut microbiota composition affecting the diversity and similarity of the bacterial community. In general, food restrictions and especially, situations of extreme food restriction plus increased activity (anorexia) seemed to have a potential negative impact on the quantity of health promoting bacteria as well as to enhance the growth of bacteria which may be related to the disruption of the gut mucosal barrier and the optimal exploitation of the very low caloric diet. On the other hand, this study highlights the associations between the gut microbiota and the appetite-regulating hormones such as leptin and ghrelin that may be important in terms of satiety control and host metabolism.
